# How far has research into disruptive innovations in the field of sustainability come to date?

**DOI:** 10.1016/j.heliyon.2024.e39134

**Published:** 2024-10-09

**Authors:** Ahmadov Tarlan, Durst Susanne, Eriksson Taina, Jussila Maria, Saaristo Aino

**Affiliations:** a Tallinn University of Technology, Ehitajate tee 5, 19086, Tallinn, Estonia; b Reykjavik University, Menntavegur 1, 102, Reykjavík, Iceland; c University of Turku, FI-20014, University of Turku, Finland; d University of Helsinki, FI-00014, University of Helsinki, Finland

**Keywords:** Disruptive innovation, Sustainability, Innovation, Sustainable development, Critical review

## Abstract

The term ‘disruptive innovation’ has been on everyone's lips for some years now, both in theory and in practice. This type of innovation is considered promising when it comes to the transition to sustainability. Based on a critical literature review comprising 121 academic articles, this study provides a systematic overview of the state of research on disruptive innovation for sustainability over 20 years. This overview makes it possible to determine whether and how an accumulation of knowledge has taken place in this area. The study reports on the selected theories, methods and definitions of the core concepts used. A thematic analysis was conducted that identified five broad themes, namely business models, people skills, supply chains, product/service development and transformations/transitions. In addition, the evolution of thematic areas over time is presented. The results indicate that there is a significant need for greater knowledge accumulation. This paper proposes concrete directions for future research to come closer to this goal.

## Highlights

• Critical review of 121 articles to map the evolution of research on disruptive innovation in sustainability context.
• Five dominant research themes could be detected.
• The themes of business models, product/service development and transition form the core of the research interests.
• Focus on people skills is more recent, while the study of value chains can be described as clearly situational.
• There is a lack of clarity, scientific rigour and the interest needed to drive the field forward in a solid way.

## 1 Introduction

Humanity is facing an urgent need to find solutions to complex problems, such as climate change and biodiversity loss [[1], [2], [3]]. Solving challenges of this magnitude requires more than minor adjustments, which is why disruptive, sustainable innovations are of interest [4,5].

The concept of disruptive innovation has been part of academic discussion since the seminal writings of [[6], [7], [8]]. Disruption has been defined as ‘a process whereby a smaller company with fewer resources is able to successfully challenge established incumbent businesses’ [9, p. 46]. Being disrupted entails that an organisation is forced to change its strategy to survive [9]. The term disruptive innovation has also attracted much attention among business professionals worldwide [10] and has become an integral part of business jargon. The word disruption seems to have degenerated into a buzzword, to the detriment of the original idea [11]. The development of work on the topic of disruption points to a growing and scattered body of literature in recent years [[12], [13], [14]]. The concept of disruption is being used quite loosely and potentially without a proper understanding of its core principles. Even in the academic literature, the concept is applied without engaging with its theoretical underpinnings or the incorporation of earlier findings. Christensen et al. [10], for example, called for meticulousness in the use of the concept in academic literature, as it is important for the accumulation of research-based knowledge and the practical applicability of that knowledge. More recently, Martínez-Vergara and Valls-Pasola [13] have argued that the definition of disruptive innovation remains vague since specific characteristics have not been identified and that, therefore, the theory needs further development. Such a situation can lead to the danger of trivial findings, discussions and studies that are pursued with little (or even no) novelty. This could prevent the field from advancing.

In addition, sustainability is a multidimensional concept that touches on a multitude of phenomena. Sustainability is traditionally understood as an orientation toward social, ecological and economic responsibility, the triple bottom line [15]. In this vein, sustainable disruptive innovation supports positive ecological, economic and social conditions and conveys characteristics of disruptive innovation [16].

To respond to the above-mentioned ambiguities in academic discussions, we propose a critical review of the existing literature on disruptive innovation in the context of a highly topical and increasingly business-critical phenomenon: sustainability. A focus on disruptive innovation seems particularly relevant in the context of sustainability, as it has the potential to drastically change the behaviour of people and organisations through the novel value dimensions and applications of the technology introduced [17]. This could bring societies closer to the transformation into sustainable societies [18], while Mäkinen [16] sees the potential for scalable, impactful solutions in this regard.

Existing literature reviews have analysed the topic of sustainability in the disruptive innovation domain. For example, reviews by Iñigo and Albareda [19] and Kivimaa [20] can be mentioned. Iñigo and Albareda [19] offer, based on their review, a framework to conceptualise sustainable innovation through operational, collaborative, organisational, instrumental and holistic components and three implementation models (self-reinforcement, self-organisation and emergence). This review, however, includes all types of sustainable innovations and hence does not paint a clear picture of the defining features of disruptive sustainable innovation. Kivimaa [20] focused on disruptive innovation as well but strictly in the transitional literature. In addition, a recent review by Datta and Srivastava [21] clarifies the concept of disruptive innovation on a general level. Their review shows the differences between disruptive, radical and breakthrough innovation and emphasises the distinguishing features of disruptive innovations in the outcomes of the innovation. In conclusion, even though some literature reviews have been published, these works have focused primarily on specific aspects, but not on the development of research in this area over a long period.

However, considering that research on disruptive innovation can be considered a field of research that is no longer in its infancy, in this paper, we try to map how research in the field has developed over the years – that is, to establish how far research has come so far. More precisely, our critical review aims to (1) enlist the theoretical and methodological choices of existing disruptive (business) innovation research in the context of sustainability, (2) elucidate the scope (theme and evolution) of disruptive (business) innovation research and (3), based on the findings, provide prospective scholars avenues to advance disruptive (business) innovation research. The focus of the review has been on scientific publications over the last 20 years (i.e., 2002–2022).

We believe that our paper contributes to the existing research on disruptive innovation related to sustainability by providing detailed information on the evolution of this field of research, thus improving our understanding of the current state of knowledge. In our opinion, there is an urgent need for a solid (and mature) knowledge base to provide a crucial foundation for improved practices in organisations in terms of disruptive innovation in a sustainability-focused context.

The paper is organised as follows. In the next section, the concepts of disruptive innovation and sustainability are outlined. Section three then presents and describes the methodology used for the review. Section four presents the findings and section five discusses them. In the final section, the conclusion and implications of the study are outlined.

## 2 Positioning of the study

As opposed to innovation, which drives continuity in the current status of matters, disruptive innovations modify development trajectories and are best understood as processes [22]. Disruptive innovations can shake or expand an existing market or create new markets [14]. The dynamics unfold as disruptive innovation introduces discontinuity through novel value attributes that meet customer needs in new ways, while incumbent actors prioritise innovation that builds continuity [10].

The theory of disruptive innovation is not without criticism, and its applicability is being questioned. However, the theory has proven useful for better understanding and explaining various shocks in the industry [23,24]. As technologies tend to develop faster than customers’ needs, continuity-driving innovation has led incumbents to overserve many customer segments [8]. King and Baatartogtokh [23] argue that incumbents would have the capacity to respond to disruptive innovation, but that they often fail in implementing and therefore end up struggling [23]. Moreover, the theory of disruptive innovation proposes that the three key factors in disruptive innovation are simplifying technology, the innovative business model and the coherent value network, in addition to which regulation also plays a role [8].

Kuokkanen et al. [25] argue in their literature review that the existing literature on sociotechnical transitions emphasises technology at the expense of business models and user understanding, thus ignoring important aspects behind the transitions, while research on disruptive innovation does not pay enough attention to the systemic nature of the phenomenon.

The literature also emphasises that no innovation is inherently disruptive, but the decisions made in connection with the innovation can be classified as disruptive [10]. According to the mapping of the existent literature by Ref. [22], the disruptiveness of innovations arises through three dimensions: technological features, marketplace dynamic and external environment. Christensen et al. [8], in contrast, emphasise that technology is not indeed an end in itself but a means of making something more convenient for the customer.

Given the prevailing sustainability challenges, technology can be an essential part of sustainable disruptive innovation [25]. Kuokkanen et al. [27, p. 760] proposed that disruptive sustainable innovation as a concept focuses on ‘which existing functions are disrupted or which attributes are transformed with more sustainable ones’. Therefore, instead of focusing on the process, disruptive sustainable innovation is concerned with the content and outcome of the process [26].

Kivimaa et al. [27] found four dimensions of disruptions: 1) markets and business models, 2) regulations, policies and formal institutions, 3) actors and networks, and 4) behaviour, practices and cultural models. A recent study has shown that not all disruptive innovations lead to transitions. However, all major sociotechnical transitions result from a bundle of disruptive innovations [28].

To summarise, there is a need to explore disruptive sustainable innovation research beyond the transition literature. The existing literature reviews do not provide insight into how and on which themes research on disruptive innovations in the context of sustainability has been conducted, but this is crucial to determine whether and to what extent an accumulation of research-based knowledge has taken place.

## 3 Methodology

To present a state-of-the-art understanding of research on disruptive innovation in the context of sustainability, a systematic literature review (SLR) approach was employed. SLRs are seen as a useful tool for establishing the current state of knowledge in research areas. They employ a precisely defined search protocol to minimise bias and guarantee the replicability and comprehensiveness of the conclusions drawn [29], which, in turn, can inform the development of future research and enhance researchers’ justifications for such research by avoiding blindly repeating prior work [30].

We followed the approaches to a systematic review outlined by Tranfield et al. [29] and Kraus et al. [31] to achieve the aim of presenting a state-of-the-art understanding of disruptive innovation in the context of sustainability. The following section describes the steps of the SLR utilised in this paper.

After defining the topic of this study, we prepared a research plan with formulated research questions and inclusion and exclusion criteria for the information to be collected. To identify relevant articles, the Web of Science (WOS) database was used. WOS provides a comprehensive view of worldwide research production [32,33] and is commonly used as the sole database in similar studies due to its ability to deliver the most comprehensive results [34,35]. In addition, we conducted a manual search in selected high-quality journals based on the ABS ranking (i.e., *Journal of Cleaner Production*, *Technology in Society* and *Technological and Social Change*) to ensure that relevant articles on the topic were not missing due to the lack of coverage of these journals in the WOS database. As keywords, the following string was used by searching it in the title, abstract or keywords in WOS: *‘sustain∗’ AND ‘disrupt∗’.* These broad keywords were deliberately chosen so as not to exclude any relevant contributions due to alternative terms.

The inclusion and exclusion criteria used are shown in Table 1.

**Table 1:** Summary of inclusion and exclusion criteria.

**Inclusion Criteria**	**Exclusion Criteria**
Papers published in the period 2002–2022	Papers published before 2002
Types of documents: articles, early access or review	Books (textbooks), book chapters, conference papers, dissertations and grey literature
Written in English	Not written in English
Peer-reviewed	Not peer-reviewed
Published in Business or Management journals (using the WOS categories: Business & Management)	Published in other journals

**Fig. 1:**
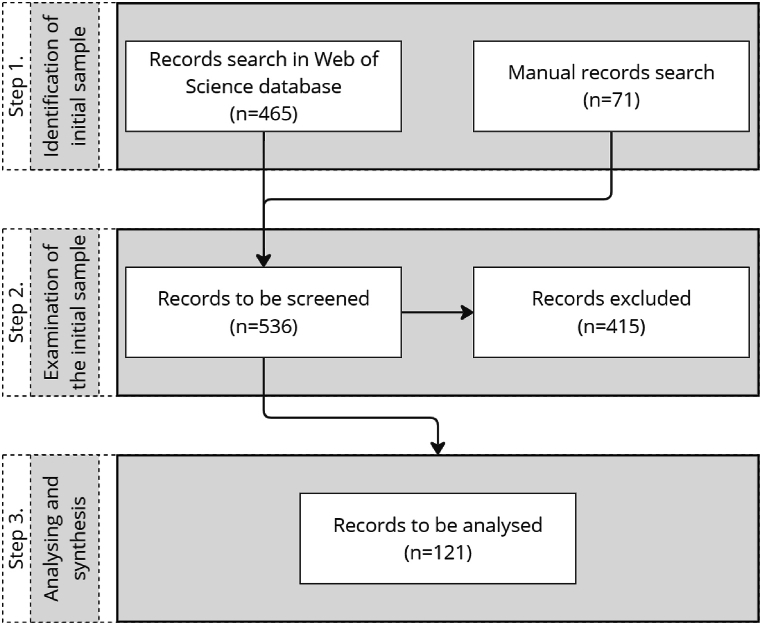
Summary of SLR steps.

Once all criteria were specified, one of us accessed WOS on May 27, 2022 to identify relevant articles. This search resulted in 465 articles. The manual searches led to the addition of 71 articles. In a first quality check, we all went through the identified articles together to weed out those articles that did not deal with the topic, that is, those that did not deal with disruptive innovation and sustainability. This was done by reading the abstracts and, if unclear, by reading further parts of the article concerned. This screening process left 121 articles to be fully read and analysed. Fig. 1 gives a detailed summary of the SLR steps taken.

An Excel table was created to facilitate the analysis. This table covers relevant aspects for meeting the study's objectives. These aspects included, in addition to the names of the authors and the year of publication, the objective of the respective article, the theories, theoretical concepts and research methods used, the definitions of sustainability and disruption used and the main results of the study.

The reading of the articles was divided among us, and each of us read the articles assigned to them and extracted the essential information based on the aspects. Once this was done, we met again and discussed the course and results of this process. This round also led to the exclusion of further papers, as we concluded that these papers did not fit the objective after all; that is, reading the paper in detail revealed that the topics of disruptive innovation and sustainability were only addressed superficially – they were used more as buzzwords. Consequently, 121 articles were left.

To illustrate the topics and their evolution, we carried out a thematic analysis. We proceeded as follows: We started with thematic open coding. This meant that each of us coded the 121 articles individually to identify possible codes that could be used for describing a paper's focus/overall direction. Once this was completed, we discussed the identified themes together in the group. If there were any discrepancies regarding the overall direction of the individual papers, we went through the article in question again together. In the next phase of coding, the goal was to bring the previously identified broad foci/overall directions to a higher, more abstract level. As before, this was initially done individually before we met again to discuss the identified themes and agree on the final ones.

This process resulted in the specification of five broad themes, namely Supply Chains, Transformations/Transitions, Business Models, Development of Products/Services and People's Skills. To add nuances to the general themes and thus make them more precise, the respective contributions were divided into sub-themes. This allowed us to better present the situation found in the respective main themes, that is, in greater detail.

## 4 Results

This section presents the results. First, the descriptive results are presented, followed by the results of the thematic analysis.

### 4.1 Descriptive analyses

The presentation of the descriptive analysis is as follows. We begin by presenting the literature used, then the theories and theoretical perspectives used and the theoretical aims of the papers before providing an overview of the definitions used in the papers covered in this review.

#### 4.1.1 Literature

We summarised the literature covered in the articles in a table (please refer to the Appendix). Given the topic and developments in the period covered by this study (e.g. the pandemic and its consequences), the particular interest in the literature on the fields of innovation and value chains is unsurprising. We also found the topics of sustainability, circular economy and risk management of greater interest in the context of disruptive business innovation.

#### 4.1.2 Theories and theoretical perspectives

The theories and theoretical perspectives applied in the articles reviewed are summarised in Table 2. It should be noted that not all the articles had a theoretical orientation, and some were literature reviews.

**Table 2:** Theories used in the reviewed papers.

**Theories/theoretical perspectives**	**Frequency**
Stakeholder perspective/theory	4
Resource-based view	3
Dynamic capability theory/perspective	3
Institutional theory	3
Complexity theory	2
Resource dependence theory	1
Communication constitutes organisation theory	1
Sociotechnical systems theory	1
Value congruence theory	1
Organisational change theory	1
Practice-based view on disruptive sustainable innovation	1
Contingency theory	1
Risk management perspective to supply chain	1
Deliberation theories	1
Systems theory	1
Behavioural reasoning theory	1
Network approach theory	1
Equilibrium perspectives of resilience	1
Practice theory	1
Expectation–disconfirmation theory	1
Regret theory	1
Foucauldian perspective on power	1
Biological ecology perspective	1
Foucault's governmentality	1
Social construction of technology	1
Talent management/HR perspective	1
Catch-up cycle theory	1
Technological paradigm theory	1
Collaborative governance theory	1
Theory of creative destruction	1
Technology-switching theory	1
Intersectionality theory	1
Transformative Consumer Research perspective	1
Legitimacy theory	1
Agency theory	1
Lifespan theory	1

**Total**	**46**

#### 4.1.3 Theoretical aims

There are different ways to contribute to theory development. For example, the theoretical aim of the paper could be to explore, develop (elaborate) or test (validate) a theory [36]. Our findings show that most of the papers included in this study emphasised development or elaboration (55 articles), followed by exploratory articles (31 articles) or articles aimed at testing or validating (23 articles). Twelve articles were assigned to other aims; that is, these papers were literature reviews or similar articles, and where the primary intention appeared to be to summarise/synthesise different aspects of disruptive innovation research or related fields.

#### 4.1.4 Sustainability and disruptive innovation defined^1^

In the following sections, we present the findings on the definitions used. The articles covered a range of sustainability issues and approached disruption in several different ways. While conducting the SLR (for details, see the methodology section above), definitions for the search terms disrupt∗ and sustain∗ were sought from each article reviewed. Sustainable and/or disruptive could have multiple different meanings in a single article. In a lack of specific definitions, the keyword was recorded with its immediate context, for example, ‘environmental sustainability’ or ‘sustainable development’. The more specific definitions found in the articles for sustainability and disruption are presented below.

##### 4.1.4.1 Sustainability

Few articles defined sustainability. More precisely, we found only 20 articles that defined the meaning of sustainability in their research. The articles cover a range of sustainability issues, for example, environmental or social sustainability, or sustainable growth. The variety of sustainability issues also means that sustainability, when defined, has different meanings.

The definitions of sustainability used are often future-oriented. Cagnin and Loveridge [37], for example, define sustainability as an organisation's ability to persist in the long-term future. Sureshchandar [39, p. 1352–1353] refers to Dyllick and Hockerts [38] in defining sustainability as ‘meeting the needs of a corporation's current direct and indirect stakeholders without compromising its ability to meet the needs of future stakeholders as well’. Sustainable growth, as defined by Allal-Cherif et al. [[39], p. 1], means that ‘business development does not contribute to reducing natural resources and endangering future generations’. This is similar to Kumari [40], who addressed sustainability in agriculture value chains and stressed that the term means that economic development and environmental and social equity needs are met without compromising the same needs in the future.

The three pillars of sustainability – economic, environmental and social sustainability – make it to many definitions of sustainability [[41], [42], [43]]. They are also notable in Bhupendra and Sangle's [44] and Wiener et al.’s [45] definitions of sustainability-oriented innovation, though the different meanings of sustainability are also covered. Turker and Ozdemir [46] note that, out of the aforementioned pillars, social sustainability is the most ambiguous and go on to develop a model for social sustainability.

Some focus on a specific strand of sustainability or sustainability in a certain context. Nijhof [49, p. 2128] defines sustainability value propositions as ‘value propositions that explicitly refer to addressing one or more – collective problems’. According to Li and Chen [[47], p. 148], firms have a sustainable competitive advantage ‘if they possess superior competency that enables them to outperform their competitors, that is not easily replaceable or eliminable, and that can be maintained over a certain period of time’. Kramar [48] defines sustainable development using the United Nation's definition. Dahlgaard and Anninos [[49], p. 466] define sustainable organisational excellence as ‘the capacity of organisations to maintain their outstanding performance – and attain long-term success by taking into consideration a balanced approach on the interests of all stakeholders – customers, suppliers, employees, shareholders, the society and the environment.’ Nissila [50] follows Garud and Gehman's [51] definition of sustainability as sociotechnical transitions: sustainable ‘niches’ come to challenge regime actors generating disruptive systemic change. Kuokkanen [17], in contrast, followed Geels [52] in their definition of sustainability transitions as systemic changes.

This emphasis on the need for balance is evident in many definitions of sustainability. Gavrila and Ancillo [53] define sustainable growth with a focus on social and environmental responsibility from a holistic perspective, whereas Gouda and Tiwari [54] refer to Kuzma [55] in defining sustainable business performance as economic, environmental and social performance. Li et al. [[56], p. 263], in reference to Pagell et al. [57], defined sustainable sourcing as ‘managing all aspects of the upstream component of the supply chain to maximize triple bottom line performance’. Johnsen et al. [58] challenge the notion that sustainable entrepreneurship is only about creating environmentally friendly and competitive products or services, but instead propose that sustainable entrepreneurship ought to change the conceptions of what sustainability is.

##### 4.1.4.2 Disruptive innovation

Most of the articles covered in this review lacked a discernible definition of disruption or disruptive, too. Disruption or disruptive were defined in 20 of the reviewed articles, despite disruptive innovation, as well as several other uses of disruption, having been employed in the articles.

Many of the identified definitions refer to other scholars who have written about disruptive innovation or disruptive technologies, which, as mentioned above, have been closely related to disruptive technologies. The influence of Christensen's work, especially, is very marked. Since disruptive innovation is a major theoretical concept for Si et al. [59], it is explained in detail, and they refer to several studies by Christensen [10,60] and others (e.g. Refs. [6,61]). Lee and Kim [62] refer to the 2013 edition of Christensen's book *Innovator's Dilemma*, originally published in 1997, in their definition of disruptive innovation as changing the rules of the game, and Wiener et al. [45] use Christensen and Raynor's [8] definition of disruptive innovation. Ferras-Hernandez et al. [63] also relied on Christensen to describe how disruptive innovations can override older innovations and companies. Turker and Ozdemir [46] define disruptive innovation as a category of social innovation that radically changes existing paradigms. Christensen's work is also noticeable in Cui et al.’s [64] definition of disruption, according to which smaller companies can challenge larger incumbents. According to Kasperovica and Lace [65], disruptive innovation is a cheaper and easier-to-use alternative to existing products that it partially or fully replaces. The simplicity of a disruptive innovation is also noticeable in Sehnem et al.’s [41] definition of disruptive innovation.

The influence of Christensen's work is considerable, and it also acts as a base for new concepts and definitions. Malodia et al. [66], for instance, address many definitions of disruptive innovation in their attempt to conceptualise reverse innovation, referring, for example, to Bower and Christensen [6] and Hart and Christensen [67]. Christensen's definition is used as a starting point for Droege's [68] definition of circular disruption, Kuhlmann et al.’s [69] definition of circular innovation and Rao's [70] definition of a frugal innovation. The original definition of disruptive innovation is challenged by Kuokkanen et al. [17], who claim that it is too limited and propose a practice-based framework for disruptive sustainable innovation.

It needs to be underlined that innovation has not been the focus of all the articles reviewed; thus, the definitions of *disruption* or *disruptive* have been slightly different. Camillus et al. [71] noted that the term ‘disruption’ has been used in many different situations and has had many different meanings. According to their understanding, disruptions ‘have potentially transformative effects on an industry’ (p. 301). Silva and Bonetti [75, p. 2] define ‘innovative technologies – as newly invented technologies that can be incremental, radical or disruptive, or existing technologies used in new ways to improve value for businesses and enhance life for humans’. Blomsma et al. [76, p. 1011] define circular disruption as ‘a transformation in a socio-technical system which causes the systemic, widespread, and fast change – to a socially and environmentally desirable and sustainable model that reduces resource consumption and addresses structural waste through the deployment of circular strategies’. Binz et al. [72] defined disruptiveness as the coexistence of or complete shift of leadership, whereas Lievens and Blazevic's [73] defined disruptive change as change induced either by digitisation or sustainability requirements. Novak et al. [74] see disruption as a synonym for shock, which works as a catalyst for change or represents a deviation from normal operations. A similar understanding is used by Prakash [75], as disruption is viewed as a random quantitative or qualitative deviation from what is expected or normal.

### 4.2 Thematic analysis

In this section, the results of our thematic analysis are presented. As mentioned in the methodology section, we grouped the papers into five broad themes: business models, people's skills, supply chains, development of products/services and transformations/transitions.

#### 4.2.1 Business models

Twenty-seven articles were assigned to the business model category. These papers were further divided into sub-themes to better show the variety of research found on this specific theme. These sub-themes are business model innovation, business model change, value creation and delivery through business models, business model enablers, types/archetypes of business models, business models discussed from higher levels and others.

Six papers focused on business model innovation and took a proactive approach to the change in business models [76]. examined business model innovation in the small- and medium-sized enterprise (SME) context and argued that structure-and process-related capabilities are important for innovating successful business models [77]. identified sociability, agility and moral inclusivity as forces that can be harnessed to overcome constraints to business model innovation, thus bringing social responsibility into the discussion. Sehnem et al. [41] found that data and digital technologies, as well as cooperation in the supply chain, are central to how firms generated market opportunities and transitioned towards circular business models. Silvestre and Fonseca [78] studied the significance of adopting an intelligence model in innovating sustainable business models [79]. found that sustainable and social goals play a significant role when a multi-sided platform organisation in the tourism sector innovates its business model. Kasperovica and Lace [65] addressed business model transformation and highlighted the need to transform all interconnected components of the business model.

Four papers assumed a more reactive approach to business model change. Miglionico [80] and Rydzak and Monus [81] examined business model improvements. Whereas the first mentioned focuses on novel technologies, the latter finds evidence of the importance of interpersonal relations, particularly cross-functional relations, for business model development. Carraresi and Broring [82] identified the importance of value chain actors in business model redesign for circularity. Jha et al. [83] stressed the importance of enablers for business model recovery: technology-related, convergence of virtual and physical space, collaboration and transparency.

Two papers focused on value creation and delivery. Si et al. [59] contribute to the understanding of how value is created, captured and delivered in sharing economy business models, whereas Glauner [84] demonstrates how the shared value concept can serve as a strategic tool in creating a business model.

Two papers had a dedicated focus on the enablers of business models. On the one hand, by focusing on the enablers of business model development, Liu et al. [85] highlighted the adoption of technological innovation. Neligan et al. [86], on the other hand, investigated the enablers of circular business models through digitalisation.

Four papers addressed different types of business models. Palmie et al. [87] and Rajala et al. [88] studied archetypes. The former, based on a set of 280 firms, showed how different utilisations of business models can be used for proposing distinct business model archetypes, while the latter identified three archetypes of closed-loop systems, namely inner circles, decentralised systems and open systems. Janssen and Moors [89] used Dutch healthcare to identify different business model types, while Munoz and Cohen [90] focused on the sharing economy.

Seven articles addressed business models at higher levels. Four of these seven articles addressed business models at the industry level: cooperative business models in the automotive industry [91], the impact of the crisis on the food and beverage industry in Bangladesh [92], business models of the Finnish food industry [17] and conditions for sustainable business models of different industries [93]. The remaining three articles addressed business models at the ecosystem level. More precisely, the relevant components of well-functioning systems were covered. Marcus et al. [94] suggested venture capital investments as a promising path for bringing forward clean energy technology. Weigelt et al. [95] stressed the importance of considering niche actors in business model activities involving multiple partners and perspectives, and Allal-Cherif et al. [39], in a similar vein, investigated the benefits of developing long-term partnerships and collaborations with partners from public and private organisations using the case of Airbus.

Two papers were assigned to the sub-theme ‘others’. Nijhof et al. [96] suggested the concept of encroachment for advancing research on business models and sustainability value propositions, while a paper by Yuan et al. [97] demonstrated how absorptive capacities can improve the management of platform-based sharing business models.

#### 4.2.2 People's skills

Seventeen articles were assigned to the theme ‘people's skills' and further divided into five sub-themes: human resource management (HRM), leadership, special skills and competences, the development of these skills and competences and others.

Four papers addressed people's skills by highlighting the role of HRM. DuBois and Dubois [42] highlighted the need for strategic HRM to prepare organisations for environmental sustainability. In a similar vein, Kramar [48] stressed the pivotal need for HRM activities to improve workplace performance, while Bierema [98] and Claus [99] called for HRM-related innovation to help create a new normal after the pandemic (the former) or reinvent talent management (the latter).

Four articles addressed the role of leadership. Mckim and Goodwin [100] focused on empowerment as a critical leadership skill to contribute to individuals’ preparedness to work collectively towards different types of sustainability. Binz et al. [72] found the capability to upgrade and catch up to be relevant for successful leadership in emergent industries. The role of different types of leadership in innovation through transformational leadership [101] and the commitment of people through responsible leadership [102] were studied as well.

Five articles investigated the different skills and competences individuals should develop or strengthen to better cope with different organisational or societal challenges. Kohtala and Hyysalo [103] found differences between makers' orientations and competences to explain these people's ability to envision the future. Human potentiality, motivation and values were determined by Dahlgaard and Anninos [49] as invisible elements of organisational excellence. According to Gouda and Tiwari's [54] study, to improve the likelihood of innovation adoption in organisations, learning agility and internal communication should be stressed. A study by Kuhlmann et al. [69] reminded us of the resistance to change in conjunction with innovation (here called circular innovation), while Sousa and Wilks [104], who focused on critical sustainable skills for the world of work in the digital age, identified the following skills: complex problem-solving, critical thinking, creativity, people management, coordinating with others, emotional intelligence, judgment and decision-making, service orientation and negotiating and cognitive flexibility.

Two papers stressed specific aspects related to people's skills and development. O'Brien et al. [105] underlined the need for learning in the area of disaster preparedness, which is expected to contribute to a more holistic approach to disaster management in organisations. Bhalla [106] showed the impact of economic benefits and perceived sustainability on people's attitudes towards collaborative consumption.

Finally, Johnsen et al. [58] argued in their paper for using the concept of style to improve social practices in entrepreneurship, while Evans et al. [107] proposed a competence framework for realising improved global work initiatives.

#### 4.2.3 Supply chains

Thirty articles discussed disruptive innovation or disruptions related to sustainability from the supply chain perspective, and these can be subcategorised into four. The first category is technology and innovation-enabled supply chain management, which focuses on how technology and innovation can be used to improve supply chain efficiency and effectiveness. The second category is resilience and risk management, which covers the strategies and practices that organisations can implement to mitigate supply chain risks and increase resilience. The third category is coordination and collaboration, which explores the importance of collaboration and coordination among supply chain partners to enhance performance. The fourth and final category is supply chain disruptions and crisis mitigation strategies, which focus on the various strategies and practices that organisations can adopt to deal with supply chain disruptions and crises, such as COVID-19.

Seven articles discussed technology-and innovation-enabled supply chain management practices. Mavi et al. [108] evaluated the existing literature on innovations in freight transport, including during crises like COVID-19, and highlighted key research themes and methodologies. Kumar and Barua [109] identified sustainable dimensions and disruptive technologies for implementation in the petroleum industry's supply chain, suggesting that AI and big data could enhance operations sustainability. Dwivedi and Paul [110] developed a framework for digital supply chain adoption from the perspective of the circular economy and highlighted the lack of digital skills and facilities as the most significant barrier to DSC development. Laforet and Bilek [111] investigated blockchain's potential use in supply chains, identifying traceability and communication as the main incentives and interoperability as the main obstacle. Xiao et al. [112] explored the relationship between technology uncertainty and supplier involvement. Ali et al. [113] investigated blockchain opportunities and challenges in the halal food supply chain and proposed a framework to overcome the challenges facing the halal food supply chain. Kamble et al. [114] presented a sustainable digital twin implementation framework for supply chains based on a review of 98 research papers.

Eight articles explored various aspects of resilience and risk management in the supply chain. Singh [115] discovered that CSR practices positively impact supply chain risk management practices and are necessary for improving the corporate reputation of organisations. Kazancoglu et al. [116] determined the enablers of resilience in FSCs during COVID-19, ranking readiness, collaboration with stakeholders and IT alignment as the most important enablers. Novak et al. [74] suggested adopting a complexity-based perspective to advance theory and practice in supply chain resilience by addressing the importance of scale and the challenges associated with mismatched scales. Loh and Thai [117] presented a management model to address port-related supply chain disruptions, aiming to minimise the potential for identified threats through practical management models to increase port resilience and maintain supply chain sustainability. Choudhary et al. [118] explored how reshoring impacts the resilience and sustainability of a focal firm's supply network. Pla-Barber et al. [119] depicted how the pandemic affected GVC configuration by driving a trend towards a more regional footprint in industries in which resilience and reliability are critical. Wasan et al. [120] constructed a value chain framework for assessing risks in lending to agro- and food-processing firms. The authors proposed it for use in conjunction with existing risk assessment models to improve the quality of credit decisions. Fasan et al. [121] tested whether companies that use green supply chain management practices benefited from a buffer effect in the context of COVID-19 and showed that these companies experienced fewer negative abnormal stock returns during the crisis.

Five articles focused on various aspects of supply chain coordination and collaboration issues. Soundararajan et al. [122] proposed a framework for developing agile sustainability governance mechanisms and explored the challenges and factors enabling authentic dialogue in emerging markets. Sharma et al. [123] analysed web conversations about supply chain challenges during COVID-19, the strategies that were implemented and provided strategic recommendations for rebuilding the supply chain. Agrawal et al. [124] reviewed coordination issues in the retail supply chain. Morsing and Spence [125] investigated the impact of large firms pressuring SME suppliers to make their implicit corporate social responsibility (CSR) communication more explicit. Finally, Li and Chen [47] presented a theoretical framework for firms with limited resources to maintain and improve their performance by improving risk management capabilities through supplier collaboration at the operational level.

Ten articles discussed supply chain disruptions and crisis mitigation strategies. Devi et al. [126] identified 10 social challenges caused by the pandemic and proposed mitigation strategies to overcome them, while Kumari et al. [40] focused on the impacts of uncertain behaviour on agricultural value chains during highly disruptive events. Montoya-Torres et al. [127] aimed to map the recent literature on logistics and SCM related to the pandemic and to provide a taxonomy and framework to support decision-making processes for logistics and supply chain professionals. Other studies [i.e., [43], [56], [75], [[128], [129], [130], [131]]] addressed issues such as disruptions in the dairy supply chain, sustainable supplier selection and the determinants of supply chain sustainability during turbulent situations. The results of these studies provide insights and guidance for industry and researchers to develop decision-support systems and improve supply chain resilience and sustainability in the face of future disruptive events.

#### 4.2.4 Developing products and services

Altogether, 15 articles investigated the development of products and services. These studies can be grouped into three subcategories: stakeholder perspective on sustainability and innovation, different types of innovations and innovation culture.

Four papers approached the development of products and services from the stakeholder's perspective. Santamaria et al. [132] developed a framework for user involvement in design practices to identify cultural codes and references for environmentally sustainable product–service systems. Lievens and Blazevic [73] focused on stakeholder engagement and, by integrating service design with B2B innovation theory, developed a granular view of stakeholder engagement. Cui et al. [64] analysed consumers' intentions and found that personal innovativeness moderates the relationship between dissatisfaction with incumbent products and intentions to purchase disruptive alternatives. Kahupi et al. [133] found that while customers are keen to be involved in sustainable innovation, investors are the most doubtful, and thus it is critical to make sure the business case is well developed. Moreover, two papers examined stakeholder-related aspects through human brands in digital news media products [134] and digital human-based solutions in the fashion industry [135].

Six papers focused on different types of innovation. For example, Rao [70] and Hall and Martin [136] examined radical technologies or innovations that aim for a more sustainable future. The latter study demonstrates the tendency of frugal innovation to disrupt industry incumbents. Malodia et al. [66] focused on factors that influence the feasibility of reverse innovation and built a conceptual framework. Inigo et al. [137] examined open innovation and argued that open innovation, coupled with alliance capabilities, can yield positive sustainability outcomes. Braganza et al. [138] studied the challenges of sustained (i.e., continued) innovation in incumbent organisations and highlighted the importance of being ambidextrous. Finally [139], examined digital servitisation as a form of innovation and found that various capabilities in relation to all dimensions of sociotechnical systems are relevant for digital servitisation.

Five papers addressed innovation culture. Dey et al. [140] argued for establishing innovation as a culture at the organisational level to advance process innovation, whereas Wiener et al. [45] stressed the significance of open foresight work in enhancing sustainability-oriented innovation. Bhupendra and Sangle [141] and Bhupendra and Sangle [44] approached innovation culture through the concept of innovativeness and distinguished between risk innovativeness and strategic innovativeness that could lead to behavioural innovativeness, product innovativeness and business process innovativeness. Finally, Heldeweg [142] investigated the role of legal regimes in experimentation, which can be an important manifestation of innovation culture.

#### 4.2.5 Transformation or transition

Thirty articles were assigned to the category of transformation/transition. These articles covered a range of changes – some disruptive – that have occurred in organisations (9 articles), industries (4) or systems (10). Five articles were categorised as primarily theoretical, while two articles were mainly about technological changes.

On an organisational level, digital transformation was the focus of four articles. Ano and Bent [143] explored how human and cultural resources affect the implementation of digital transformation strategies in multigenerational family businesses. Digital transformation was also the focus of Rossini et al. [144], who investigated the role of lean production in digital transformation. Lee and Kim [62] looked at the case of a mobile operator and how it used digital technology to expand to a new smart energy business, while Gavrila and Ancillo [53] studied how organisations could shift their business model towards sustainable growth after the COVID-19 pandemic and how this can be achieved through entrepreneurship, innovation, digitisation and digital transformation.

Three articles focusing on transformations in organisations were more theoretically oriented. Cagnin and Loveridge [37] developed a framework to enable business networks to evolve towards sustainable development, while Bhargava [145] demonstrated how organisations can use virtuality as a starting point when confronting disruptive challenges. Camillus et al. [71] aimed to offer a strategic management approach to organisations faced with societal disruptions.

Two more articles were assigned to the subcategory of organisations. Sureshchandar [146] was interested in Quality 4.0 implementation in organisations and Ray [147] in the sustainability challenges of an oil company. The focus of Ray's [147] work was more specifically on sustainability strategies for large-scale projects.

Four articles were assigned to the subtheme of (disruptive) transformation at the industry level, and thus some of them represent the most ‘traditional’ way of understanding the effects of disruptive innovations. The sources of disruption could be new technology [46,63,148] or the business sector and the way it operates [149]. Lekan et al. [148] researched disruptive innovations in the construction field to achieve sustainable development goal 9, sustainable infrastructure. Throop and Mayberry [149] were interested in industry disruptions, which they approached with ‘sustainable virtues’. Ferras-Hernandez et al. [63] studied how start-ups that control key technologies threaten the foundations of the automotive industry, and Turker and Ozdemir [46] investigated the hospitality industry, differentiating between disruption and transformation, as they found that one of the companies they studied had transformed its industry, while the other disrupted the whole system.

Systemic transformation or transition happening on multiple levels was the focus of 10 articles. Matschoss and Heiskanen [150] focused on local sociotechnical transition pathways from a multilevel perspective. Nissila [50] studied the role of conferences as arenas for creating new, sustainable fields and found that conferences have had a role in generating pathways to disruptive systemic change.

A subset of systemic transformation has been categorised as circular economy and circular disruption, which have been the focus of five articles. Bai et al. [151] explored the impact of Industry 4.0 technologies on social sustainability with a circular economy approach. Garcia-Quevedo et al. [152] focused on the barriers to the circular economy that European SMEs face. Yazdani et al. [153] discussed the implementation of circular economy strategies in agriculture and the effects of disruptions on supply chains.

Two articles were interested in circular disruption. Blomsma et al. [154] proposed a framework for circular disruption consisting of three core phases of disruption, whereas Droege et al. [68] looked at circular economy policies and how they can support circular disruptions.

Three articles focused on urban transformation. Two of them included an element of a sharing economy– both studies used the case of bike-sharing in Shanghai [155,156]. The third article consisted of three case studies of systemic disruption that were achieved through urban intervention in New York City [157].

Five articles were more theoretical or dealt with conceptualisations. Den Hond and Moser [158] argued for a more nuanced way of talking about technology, thus disrupting the understanding of technology. Similarly, Garvey et al. [159] questioned the discourse, claiming that new green technologies in the agrofuel sector disrupt old power imbalances. De los Reyes and Scholz [160] called into question the capacity of ‘creating shared value’ to eventually eradicate ecologically destructive businesses. Spotswood et al. [161] explored the effect of practice theory on traditional social marketing approaches, and Schiavone et al. [162] reviewed the digitalisation of manufacturing systems towards sustainability.

Finally, two articles investigated technological changes. Khan and Bohnsack [163] studied how the design of the value proposition influences the disruptive potential of sustainable technology, and Phirouzabadi et al. [164] investigated knowledge mechanisms within powertrain technology.

#### 4.2.6 Evolution of the research themes

**Fig. 2:**
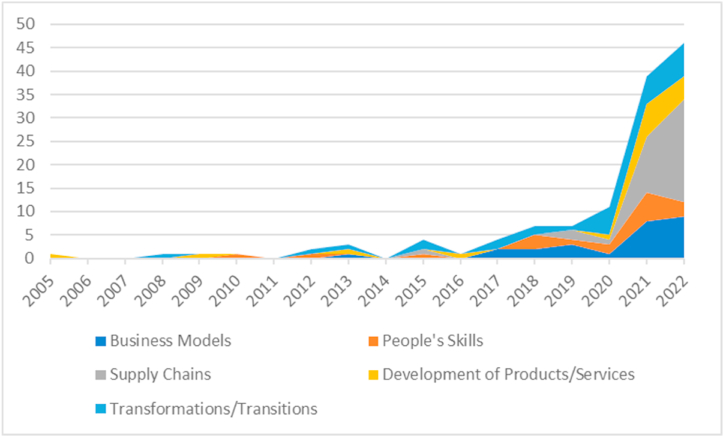
Evolution of the research themes.

The evolution of the research themes can provide valuable insights into how the landscape has transformed over the years, particularly in response to disruptive events such as the COVID-19 pandemic. Hence, such a descriptive analysis enables a deeper understanding of the changing priorities and trends that have shaped the field over the past decade. Looking at the annual distribution of articles across the different research topics presented earlier, it becomes clear that these topics have gone through different development paths (Fig. 2).

In the following section, we analyse the evolution of each theme based on descriptive statistics.

The theme of business models has seen a remarkable increase in interest and research, particularly during, 2022 and 2021, accounting for more than half of the articles (nine and seven articles, respectively). The remaining 14 articles were published in the preceding years, with three articles in 2019, two articles each in 2018 and 2017, and two articles in 2013. This increase can be linked to the particular challenges posed by the COVID-19 pandemic and an increased interest in sustainability issues, forcing organisations to adapt and innovate. Organisations were (and are) looking for resilient and innovative business models to be prepared for uncertainties. This shift towards adaptable models not only emphasises survival but also a more sustainable approach to business operations. Sustainable business models that prioritise long-term viability and societal well-being have gained prominence, as companies recognise the need to balance profitability with environmental and social responsibility.

The concentration of publications on the theme of Development of Products/Services, especially in 2021 with seven articles, followed closely by 2020 with three articles, signifies a heightened emphasis on innovation and customer-centricity. Two articles were published in 2022, while the remaining articles were distributed across previous years, including one article each in 2017, 2016, 2013, 2009 and 2005. This aligns with businesses’ responses to changing consumer demands during the pandemic.

The increased research attention on people's skills was recorded in 2022, 2021 and 2020 with two articles published in each of these years, while 2019, 2018, 2015, 2012 and 2010 each saw the release of one article. These findings suggest a consistent interest in exploring and understanding people's skills, with a slight increase in research output in recent years.

The theme of supply chains has experienced a significant surge in publications, particularly in 2022 and 2021, with 13 and 12 articles, respectively. In 2020, only one article was published, while 2019 and 2015 witnessed the release of three articles and one article, respectively. The disruptions caused by the pandemic exposed vulnerabilities in global supply chains, prompting a re-evaluation of supply chain strategies for resilience and sustainability. The urgency to ensure the availability of essential goods and services underscored the need for robust logistics and sustainable supply chain practices. These results indicate a substantial focus on the supply chain domain in recent years, emphasising the importance of this area in contemporary research.

Interest in the transformation/transitions theme has been consistent over the years, with a peak in research activity from 2020 to 2022, with seven articles in 2022, closely followed by 2021, with five articles. The year 2020 saw the release of seven articles as well, while 2019, 2018, 2017, 2015, 2013, 2012 and 2008 each had one or two articles published, which reflects an enduring focus on organisational adaptability amidst changing market dynamics. This theme's sustained relevance suggests an ongoing need for businesses to navigate transitions effectively.

#### 4.2.7 Evolution of research methods with regard to the themes

**Fig. 3:**
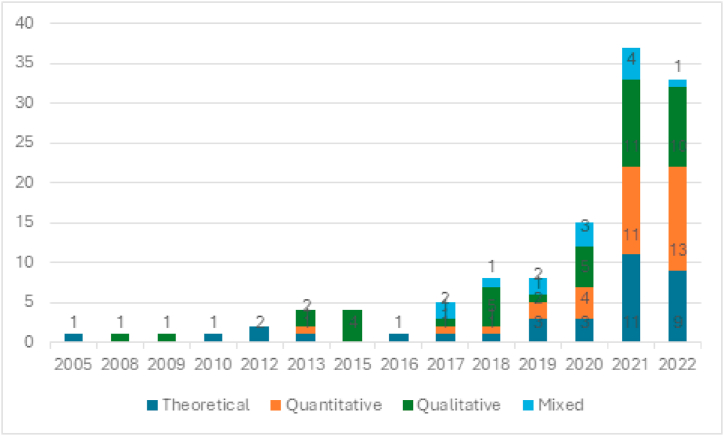
Evolution of the research methods.

To map the development of research in the selected period, we also analysed which research methods were used in the various themes over the years. Fig. 3 details the evolutions of the 121 articles used, showing the increase in theoretical-focused articles, particularly since 2019. The number of mixed-method articles remains relatively low, with a slight increase in recent years. Moreover, the consistent presence of qualitative studies across the years suggests the enduring relevance of qualitative methods in generating rich, contextually grounded insights. The substantial increase in qualitative articles since 2018 implies an even stronger interest in qualitative research methods. At the same time, the results indicate that quantitative research methods remain the dominant approach in the field.

The following section explains the methodological focus of the articles on individual broad themes. The results indicate that the research methodology in the studies focusing on the theme of business models has changed considerably over the years. While quantitative research methods such as surveys (data collection) and regressions (data analysis) have been increasingly used in previous years, a shift towards qualitative methods in the form of more case studies and grounded theory approaches for researching disruptive innovations and business models can be observed in recent years, especially from 2018 onwards. In some studies, newer methods, such as multi-criteria decision-making (MCDM) and qualitative comparative analysis with fuzzy sets (fs/QCA), were also used to expand or deepen knowledge in this subject area.

Regarding people's skills, researchers have employed different research methods to explore this theme. In the early 2010s, theoretical frameworks dominated the discourse, and by 2015, empirical studies began to gain prominence. The transition to empirical research accelerated in 2018, with a surge in studies using self-reported questionnaires (data collection) and structural equation modelling (data analysis). The empirical turn continued into the 2020s, with researchers combining quantitative and qualitative approaches. Some theoretical works have aimed to contribute to the development of the theme, for example, by introducing stakeholder dynamic capability in this regard.

The topic of supply chains has been examined using various quantitative and qualitative research methods, such as interviews, case studies, surveys and optimisation models, which not only suggest different research questions and perspectives but also efforts towards theory development. Additionally, the adoption of novel research methods, such as the hesitant fuzzy set from 2022, suggests the willingness of certain researchers to use different ways to explore supply chain-related topics. There are also literature reviews aimed at synthesising the current body of knowledge in selected supply-driven areas. The use of different methods also indicates that this is a saturated research field (theme), and novelties are seen, in particular, in the use of alternative methods, such as those that go beyond surveys, interviews and case studies.

The study of the theme development of products and services has been a subject of both empirical and theoretical research methods, with a noticeable shift from primarily theoretical research in earlier years (2005, 2013, 2016, 2017) to a focus on empirical research in later years (2020, 2021, 2022). Empirical studies in this area have employed quantitative methods, such as experiments and surveys, as well as qualitative methods, such as qualitative case studies, to investigate, for example, the factors that contribute to successful product and service development. Theoretical works in the form of literature reviews and concept development have also been utilised by some researchers to further or synthesise the understanding of this theme.

The theme of transformations/transitions was investigated using a variety of methods and thematic focuses. In earlier years, such as 2008 and 2013, empirical research was based mainly on case studies. Over time, the use of empirical methods has expanded to include various techniques and data, such as quantitative data analysis, bibliometric data, semi-structured interviews and field observations. Meanwhile, theoretical work has been used to propose frameworks and models related to sustainability, social upheaval and dynamic stakeholder capabilities that facilitate the adaptation of organisations to changing environments and new challenges.

## 5 Discussion

In the following section, we discuss the findings presented in Section 4. The structure follows the sequence presented above.

### 5.1 Literature and definitions

The findings demonstrate that the papers analysed in this review base their works primarily on the supply chain or innovation literature. The focus on the former is not surprising, given the background of the pandemic and its consequences for national and global value chains and, therefore, the large number of papers that have been published on the subject in recent years. At the same time, the large number of articles that have their starting point in the innovation literature is not surprising, since that is the basis of the disruptive innovation concept. One would even have to expect this if one assumed that the contributions were based on existing knowledge. Other articles have their starting points in the literature on sustainability, circular economy and risk management – areas of literature that have also come to the fore strongly due to recent developments.

If one takes a closer look at the applied literature, for example, the innovation literature, one notices that only a small minority of the studies reviewed explicitly build on the disruptive innovation literature. In addition, the lack of definitions for key concepts is a serious shortcoming that undermines the accumulation of knowledge. Due to the fragmented nature of the literature, the reviewed papers in this area do not help to develop a comprehensive understanding of sustainable disruptive innovation. A similar tendency can also be seen in the articles positioning themselves in other literature. Again, the forthcoming literature seems to be applied rather superficially, and the focus seems to be more on the use of mainstream topics/terms around which a paper is developed.

As many of the articles lacked definitions for either or both disruption and sustainability, we recognise that these terms are often used as buzzwords, possibly to generate interest in the article without engaging in depth with previous findings and insights. This handling of terms could also be due to their everyday use or daily presence. In research, however, an imprecise use or lack of definition/description of the core concepts is tricky as it raises the question of what has been studied at all. It also questions the reliability and validity of the findings presented. Due to the lack of definitions in the majority of articles, it is difficult to evaluate how well the studies covered in this review built on extant knowledge. Additionally, evaluating the coherence of the conceptual approaches of the studies is a significant challenge when key concepts are not properly defined.

### 5.2 Theories and theoretical aims

Given the sporadic work with definitions, a similar pattern in the use of theories or theoretical models is not surprising. Some theories are more commonly used, such as the stakeholder perspective/theory or the resource-based view. Otherwise, the picture that emerges is more like a collection of different theories mentioned by the respective authors. However, a direction or specific directions cannot be read.

In terms of the theoretical aims, the review suggests that the field has started to take off, with most articles interested in topic development and/or elaboration. There is also an increasing number of papers that tested constructs/theories and so on. Against the background of the situation described above, however, the question arises as to whether the field should not take a step back and work more carefully with the theoretical development and consolidation of the topic, specifically the core concepts and their definitions and conceptualisations.

### 5.3 Methodology

The development of the research methods applied in the proposed themes indicates a shift from primarily quantitative methods to a broader spectrum of different research methods.

The shift from quantitative to qualitative research methods in the field of business models suggests that actors in the field have learned about the relevance of qualitative research for developing theory – that is, advancing a field of study. The theme of people's skills also benefits from a variety of different research methods. Only then can researchers gain insights into different aspects of people's development and qualifications that are important for disruptive innovations based on sustainability. Interest in the supply chain topic has generally increased significantly due to recent developments. It is also positive that research in this area has become broader, and researchers are using new/different methods to better address the chosen research objectives. A higher (broader) methodological competence can also be observed in the other two themes. This situation could also indicate that methodological training at universities has improved both in terms of breadth and depth.

### 5.4 Research themes and directions

The articles under the business models theme bring forth several relevant insights. Business model innovation guided by sustainability objectives is linked to novel capabilities as organisations need to start managing platforms or overcome constraints. The reviewed studies also highlight the systemic nature of business model innovation, for example, in circular or sharing economy contexts. In addition, the role of interpersonal and interorganisational relations are notable topics in the reviewed studies focusing on business models. The articles also open the discussion about novel and alternative approaches to business models to create sustainable companies around disruptive innovations (see also [165]). However, the resulting picture remains unclear; rather, it is a scattered collection of individual studies.

The people's skills theme also deals with interpersonal relationships, especially concerning the individual actor. Some of the articles focused on the skills required to meet the demands of working with disruptive innovation for sustainability and how these skills can be developed. However, to date, there does not seem to be a coherent understanding of core skills or abilities. Some studies stress leadership skills. Many papers emphasise, at the organisational level, the importance of adapting the role of HRM in this context, as there is a need to shape employee mindsets amidst change.

In the thematic area of supply chain studies, it was found that questions about sustainability are more important than concrete considerations about disruptive innovations. Several studies integrated resilience, risk management or risk reduction in supply chains with sustainability aspects. There was a focus on sustainably securing the supply chain, which can be linked to recent developments. In addition, the role of coordination was present in many studies, either through collaboration or digital interaction and the use of data.

As expected, studies focusing on product and service development often emphasise the stakeholder perspective and examine, for example, the involvement of different stakeholders as part of the development process. This category includes studies on different types of disruptive innovation, such as frugal, reverse and open innovation. Although this is welcome, these studies are still isolated examples that take one approach, so deeper knowledge of this has not yet been accumulated. There are also studies in this thematic area that shed light on the challenges for established companies in the sector regarding sustainable disruptive innovations (product/service development).

Compared to the other themes, the theme of transformation/transition can be described as fragmented, which is reflected in the 10 identified sub-themes. On the one hand, this indicates that the state of knowledge in this thematic area is still in its infancy. On the other hand, it makes the accumulation of knowledge quite difficult. The diversity found may also indicate that transformation topics are relevant in many different research directions.

On a higher level, it can be said that three (i.e., business models, product/service development and transitions/transformations) of the five research themes we identified tend to focus on the disruptive innovations and the transitions they require. While papers in the thematic areas of supply chains and people's skills deal with the concept of business disruption on a different level, the former takes a narrower view, while the latter looks at the issue on a broader level. For example, it is obvious that when addressing competences for disruptive business innovation in terms of sustainability, specific competences are addressed and emphasised, which in turn need to be linked to the other competences expected of an employee/manager to be able to cope with current and future challenges in the company. Similarly, vulnerable but systemically relevant supply chains must ensure that they can continue to function sustainably, even after an incident. Hence, research activities in these two thematic areas require a broad and comprehensive perspective.

The analysis of the main themes has also shown that a topic such as disruptive innovation is taken up by mainstream research and driven by current events (e.g. crises), which explains, for example, the strong focus on value chains and related topics. However, given the situation described above, which emphasises that theories are hardly used or neglected, the question arises as to what kind of knowledge or contribution is produced in the articles. Who is interested in results where a rigorous scientific approach is not applied or is applied only incompletely? For which target groups are the authors in question writing? To which target groups does the research field of disruptive corporate innovation for sustainability appeal?

## 6 Conclusions, Suggestions for future research and limitations

Based on a critical literature review, this paper illustrates how research on disruptive innovation in the context of sustainability has evolved over the years. As far as we know, this study is the first to aim to summarise the state of knowledge from the period 2002–2022 and to show that the concept is more than just a buzzword. More precisely, we wanted to establish how mature the field of investigation is.

Based on the review, it can be stated that the study of disruptive innovation in conjunction with sustainability has addressed many different aspects of the covered period. In particular, we see a clear need for more exploratory studies to advance theory development and strengthen the theoretical framework of the field. This applies to the field as a whole, as well as to individual themes and new themes. The current state of play suggests that there is an urgent need to demonstrate scientific rigor and focus to accumulate existing knowledge and arrive at a more holistic understanding of disruptive innovation in the sustainability context.

In more detail, the presented review shows that there is no clarity in terms of the theories and literature on which studies in this area are built. Instead of using single established theories, a wide variety of different theoretical approaches have been applied. This makes it difficult to make statements about relevant and appropriate theoretical approaches and perspectives. The examined articles also do not make it easy to understand what the authors mean by the core concepts. This, in turn, makes it difficult to evaluate these articles in terms of their contribution to knowledge accumulation and deepening.

Based on the findings and assuming that the topic is relevant to making an active contribution to sustainability, we see a particular need to consider the following points in future research.

● Future research should be clear and rigorous in how the key concepts, specifically disruptive innovation and sustainability, are defined and used in the studies; for example, researchers should outline and justify how disruptive innovation is measured or operationalised.
● Future research should focus on theory-driven research. The applied theories and their suitability for the study at hand should be thoroughly discussed and justified. Adding a new theory or theoretical perspective that has not been used so far does not, per se, contribute to a better understanding of the topic.
● In addition to theoretical work, there is also a need for additional empirical work. For example, replication studies could be carried out to verify the robustness and generalisability of previous results and thus contribute to cumulative science.
● Future research should show whether and how existing knowledge and understanding have been incorporated into their studies to promote knowledge accumulation. If this were not done, it would be interesting to know why the researchers chose not to do so. Possibly challenging existing knowledge (conventional wisdom)?
● Future research needs to address the complex systemic nature of sustainability in the domain of disruptive innovation. For example, collaboration within and between organisations is highlighted in multiple themes. The same refers to the important role of digitalisation, the use of data or novelty in value added. However, existing studies do not address these complexities comprehensively. Therefore, studies building on a solid theoretical foundation and inspecting the phenomenon from multiple perspectives are needed. The themes and subthemes identified in this review could form the basis for studying disruptive innovation for sustainability from different perspectives and angles.
● Future research could also be conducted to demonstrate the role of disruptive innovation in reaching the UN's sustainable development goals. For example, how does disruptive innovation contribute to responsible consumption and production (goal number 12)? Another example could be investigating collaborative disruptive innovation activities dedicated to sustainability as a possible contribution to Partnerships for the Goals (goal number 17).

More rigorous research activities in this area would also make an important contribution to the perception of the field among practitioners. Entrepreneurs and managers, for example, would gain trustworthy insights into the relevance of disruptive innovations to a firm's sustainability efforts. Politicians would receive the necessary information to develop and implement more targeted investment and subsidy programmes.

As with any research, this review has limitations. The review process chosen in this paper may not have allowed us to identify all relevant articles in the field of disruptive business innovation in the sustainability context. This refers to the search terms and the inclusion and exclusion criteria. Furthermore, based on the findings, only a few research directions can be proposed. The field offers many more opportunities for research.

We, the authors, hope that this study provides a solid starting point for further rigorous research on disruptive innovation and sustainability.

## CRediT authorship contribution statement

**Ahmadov Tarlan:** Writing – review & editing, Writing – original draft, Methodology, Formal analysis, Data curation, Conceptualization. **Durst Susanne:** Writing – review & editing, Writing – original draft, Supervision, Methodology, Formal analysis, Data curation, Conceptualization. **Eriksson Taina:** Writing – review & editing, Writing – original draft, Supervision, Methodology, Formal analysis, Data curation, Conceptualization. **Jussila Maria:** Formal analysis. **Saaristo Aino:** Writing – review & editing, Writing – original draft, Formal analysis, Data curation, Conceptualization.

## Data availability

The data used in this paper are referenced.

## Funding

This research did not receive any specific grant from funding agencies in the public, commercial, or not-for-profit sectors.

## Declaration of competing interest

The authors declare that they have no known competing financial interests or personal relationships that could have appeared to influence the work reported in this paper.

## Figures and Tables

**Table:** 

Literature	Number of articles
Absorptive capacity	1
Agility	1
Alliance-management capability	1
Ambidexterity	1
Attitude and motivation	1
Biodiversity	1
Blockchain	1
Bottom of pyramid	1
*Business model*	*5*
- Business model	4
- Business model – digital transformation of a business model	1
Circular economy	10
Clean energy	1
Closed loop	1
Control mechanism	1
Cost benefit analysis	1
Corporate reputation	1
*Corporate social responsibility*	*3*
- Corporate responsibility	1
- CSR	1
- CSR practices	1
Cultural value	1
*Digitalisation and digital transformation*	*4*
- Digital transformation	1
- Digitalisation	2
- Digitalisation – digitalisation of manufacturing systems towards sustainability	1
*Disaster management*	*2*
- Disaster management	1
- Disaster management – business disaster experience	1
*Disruption*	*4*
- Disruption	3
- Disruption – disruptive potential of sustainable technologies	1
Dynamic capabilities	1
*Entrepreneurship*	*3*
- Entrepreneurship	1
- Entrepreneurship – digital entrepreneurship	1
- Entrepreneurship – social entrepreneurship	1
Environmental sustainability	1
Exclusiveness and star power	1
Field-configuring events	1
Global commodity chain – techno–institutional lock-in	1
Global value chain	1
*Governance*	*2*
- Regional governance	1
- Experimentalist governance	1
Green manufacturing	1
*Human resource development*	*3*
- Human resource development	1
- Sustainable HRM	1
- Strategic HR	1
*Industry 4.0*	*2*
- Industry 4.0	1
- Industry 4.0 – technologies	1
*Innovation*	*24*
- Innovation – adoption	1
- Innovation – B2B	1
- Innovation – business ecosystem	1
- Innovation – business model	2
- Innovation – disruptive innovation	4
- Innovation – frugal innovation	1
- Innovation – green	1
- Innovation – innovation paths	1
- Innovation – process Innovations	1
- Innovation – social innovation	1
- Innovation – sustainability-oriented innovation	1
- Innovation – systems of innovation	1
- Innovation – technological innovation	1
- Innovation – technological innovation system framework	1
- Innovation – Technological Innovations Systems (TIS)	1
- Innovation – open innovation	1
- Innovative capability	2
- Innovativeness – organisational innovativeness	1
- Innovativeness – personal innovativeness of the disruptive innovation product	1
Leadership – responsible leadership	1
Materialism	1
Network	1
Organisational excellence	1
Performance	1
Quality management	1
Reporting	1
Reshoring	1
*Resilience*	*5*
- Resilience	4
- Resilience – organisational resilience	1
*Risk management*	*8*
- Risk and disruption management	1
- Risk management	2
- Climate risk management process	1
- Risk analysis	1
- Risk identification	1
- Risk management perspective to supply chain	1
- Supply chain risk management	1
Shared value	1
*Sharing economy*	*5*
- Sharing economy	3
- Sharing economy – brands	1
- Sharing economy – business	1
Smart city	1
Smart logistics	1
Source of funding – clean energy – Venture Capital	1
*Sourcing*	*2*
- Strategic sourcing strategies	1
- Sustainable sourcing	1
*Stakeholder management*	*3*
- Stakeholder engagement	1
- Stakeholders	2
*Strategy*	*4*
- Business	1
- Cognitive strategic groups	1
- Organisational strategy	1
- Strategic niche management	1
Strength of weak ties	1
Structural holes	1
*Supply chain*	*27*
- Supply chain	2
- Cold supply chain (CSC)	1
- Digital supply chain	1
- Resilient supply chain	1
- Reverse supply chain coordination	1
- Supplier collaboration	1
- Supply disruption	1
- Port-related supply chain disruptions	1
- Sustainable supply chain	1
- Supply chain disruption	1
- Supply chain disruption recovery models	1
- Supply chain financing	1
- Supply chain management	2
- Green supply chain management,	1
- Information sharing between supply chain partners	1
- Sustainable supply chain management	1
- Supply chain network	1
- Supply chain network risk drivers	1
- Supply chain resilience	4
- Supply chain robustness	1
- Supply chain sustainability	1
- Supply chain vulnerability	1
*Sustainability*	*14*
- Sustainability	5
- Sustainability management	1
- Sustainability performance	1
- Sustainable Agriculture Value Chain	1
- Sustainable development	1
- Sustainable growth	1
- Sustainable mobility	1
- Sustainable operation	1
- Sustainable supplier selection	1
- Sustainable value proposition	1
Teams – Transition to virtual teams	1
Virtues – Sustainable virtues	1

## Footnotes

1 The search terms used having been disrupt∗ and sustain∗, some of the articles use the term disruption while others use the term disruptive, and many derivatives of sustain∗ are equally used. Here, the terms disruption and sustainability are used to convey all the different derivatives that have been used in the articles
